# Parking and Public Health

**DOI:** 10.1007/s40572-024-00465-4

**Published:** 2024-12-11

**Authors:** Michael D. Garber, Tarik Benmarhnia, Jacob Mason, Emily Morales‐Zamora, David Rojas-Rueda

**Affiliations:** 1 Scripps Institution of Oceanography, UC San Diego, San Diego, CA USA; 2 Irset Institut de Recherche en Santé, Environnement et Travail, Inserm, University of Rennes, EHESP, Rennes, France; 3 Institute for Transportation & Development Policy, Washington, DC USA; 4 Emoza LLC, Fort Collins, CO USA; 5 Department of Environmental and Radiological Health Sciences, Colorado State University, Fort Collins, CO USA; 6 Colorado School of Public Health, Colorado State University, Fort Collins, CO USA

**Keywords:** Urban Planning, Housing, Transportation, Equity, Public Health, Environmental epidemiology

## Abstract

**Purpose of Review:**

Parking is a ubiquitous feature of the built environment, but its implications for public health are under-examined. This narrative review synthesizes literature to describe pathways through which parking may affect population health.

**Recent Findings:**

We begin by contextualizing the issue, outlining key terminology, the sheer scale of land dedicated to parking, and the historical factors that led to this dominant land use. Next, we delineate four pathways linking parking with public health: 1) Promoting driving and car dependency, affecting air pollution, greenhouse-gas emissions, physical activity, traffic-related injuries and fatalities, and accessibility; 2) Creating impervious surfaces, with consequences for urban heat, flooding, water pollution, and green space; 3) Affecting housing affordability and associated health outcomes of this social determinant; and 4) Design, construction, and maintenance, the health impacts of which include on-street parking’s positive and negative impacts on safety for all roadway users, air-quality effects of parking’s construction and maintenance, and the potential for green parking lots to mitigate some health consequences of impervious surfaces.

**Summary:**

While evidence supports each pathway, additional empirical research is needed to evaluate impacts of parking on public-health outcomes. As a dominant feature of the built environment with many health implications, parking warrants attention by public-health research and practice.

## Introduction

The importance of the built environment and urban design on public health is now widely recognized [1, 2]. The early 2000s saw a rise in research by the planning and health communities investigating effects of urban design and land use on physical activity, obesity, transportation injuries, and other health-related behaviors and outcomes [1]. This and subsequent research have led to the inclusion of built-environment-related topics in federal public health objectives. For example, *Healthy People 2030*, a set of priorities by the U.S. Department of Health and Human Services, includes several objectives under the broad goal of “create(ing) neighborhoods and environments that promote health and safety” [3]. The importance of urban design on health is also reflected in global objectives. The United Nations Sustainable Development Goal 11, for example, is to “Make cities inclusive, safe, resilient, and sustainable” [4].

Despite this recognition by researchers and practitioners, car parking, a dominant built-environment feature of urban and suburban environments in the United States and many other countries, is rarely a focus of public-health literature. In the United States, estimates suggest space for parking consumes between 8,500 and 24,000 square miles (discussed further below) [5]. The lower bound of that range is about the land area of the U.S. state of Massachusetts [6].

Some recent reviews on pathways between urban planning, transportation environments, and public health mention parking [7–11]. No review article in the public-health literature, however, has focused specifically on the various potential impacts of parking on public health. Given the sheer amount of land parking occupies, its potential impacts on health are worth examining as a standalone topic. Many of the pathways that connect parking and health may be similar to those described in existing frameworks relating urban transportation and its infrastructure more generally with health (for example, by affecting transportation mode share, physical activity, and air pollution) [8], yet other health pathways may be unique to parking, such as its potential impact on housing affordability [11].

In this narrative review, we explore and summarize the existing knowledge relating parking to public health. We begin with an overview of parking terminology, estimates of the amount of land occupied by parking in the U.S., and a brief history of how parking came to occupy this much land. We then review potential pathways through which parking may affect health, organizing the review into four pathways. We conclude by offering potential directions for future health-related research of parking and parking policy.

## Car Parking Overview

### Parking Definitions

The noun *parking* may either refer to the gerund of the verb *to park* – “the act of leaving a car somewhere for a period of time”—or to “a space or area where vehicles can be left.” [12] Unless otherwise specified, we focus on the latter of these two meanings in this paper. That is, we focus on parking infrastructure [5, 13], the surface parking lots, on-street spaces, buildings, and other facilities or areas whose designated purpose is car storage.

Parking facilities can be *on-*street or *off-street* [5, 14]. On-street parking, also called curbside or curb parking [15, 16], refers to parking on the side of a public road or street [14]. On-street parking commonly takes the form of parallel parking, but can also be angled or perpendicular [17]. Off-street parking refers to parking facilities that are located away from public roads or streets. Off-street parking includes surface parking lots (a dedicated area for parking consisting of one level of parking on the ground [18]) and buildings whose purpose is car storage. These buildings can serve commercial or residential use and are variously called structured parking, parking decks, parking garages, garages, or other similar terms [19]. In American English, the term *garage* commonly refers to a residential garage, specifically, that is, a walled, roofed structure for storing vehicle(s) that may be attached to a home or a separate building [20].

### Amount of U.S. Land Occupied by Parking

Quantifying the share of land devoted to parking is important for understanding the scale of this built-environment feature and for providing context to the magnitude of its potential health consequences and opportunity costs if the land were used another way. Above, we stated that parking occupies between 8,500 and 24,000 square miles in the United States. This range is based on estimates that the U.S. has between 700 million and two billion parking spots [5, 21, 22], assuming 330 square foot per spot [16, 21]. The lower bound of 8,500 square miles equates to 0.25% of the total land area of the United States and 0.29% of the contiguous U.S.

This land-use proportion tends to be much higher in urbanized areas. In Los Angeles County, a study estimated that parking infrastructure of one type or another covered 14% of the county’s incorporated land in 2010 [13]. A similar study of metropolitan Phoenix estimated parking’s land share to be 10% [23]. A land-use percentage in the low- to mid-teens is roughly consistent with estimates by the Parking Reform Network who have used OpenStreetMap data to estimate the percent of *developable land* occupied by parking in the ‘central city’ of 53 U.S. cities at this writing [24], with estimates ranging from 1% in New York City to 42% in Arlington, Texas (average across included cities = 20.8%, median = 21%). They assume that 75% of the central city area is *developable land*. *Central city* is their term for the city’s downtown or its most central neighborhood. To compare these estimates with others expressing parking’s share of the total land area [5, 13], we can multiply their reported percents by 0.75. The average percent of total area devoted to parking of the central city of these cities is then 15.6% (median = 15.75%).

The amount of land occupied by parking in the contiguous U.S. has also been estimated by the United States Geological Survey (USGS) using satellite imagery among other sources, and a dataset is available with estimates of the percent of each county occupied by parking [25]. To find the total square area devoted to parking in each county according to this dataset, we multiplied each county’s square area by its parking land share in 2012. The sum over all counties, 14,145 square miles of parking, is within the range of 8,500–24,000 square miles noted above. Assuming 330 square feet per space, those 14,145 square miles would equate to 1.31 billion parking spots, which is within the range proposed by Chester and colleagues [5]. Using the U.S. Department of Transportation Federal Highway Administration’s reported figure of 276 million total vehicle registrations in 2020 [26], these 1.31 billion spots would equal 4.76 spots per car, similar to Shoup’s ballpark estimate of four spots per car [16]. That the figures calculated from the USGS data roughly agree with previous estimates by Shoup [16], Chester [5, 22], and colleagues is evidence of convergent validity that each are accurate to a rough approximation. While the precise amount may remain unknown, the broad point is that vast expanses of land (say, an area at least the size of Massachusetts) have been devoted to the temporary storage of cars in the United States.

### Ubiquitous Parking in the U.S.: A Brief History

The history of how parking came to occupy so much land in the United States began when off-street parking facilities were devised as an approach to move cars away from the street to ease congestion in the early half of the twentieth century [15, 16, 27]. Research in the 1920s suggested that on-street parking reduced the capacity of a typical downtown street by 30%−50% due to double parking (cars parked side by side), cruising for parking, and the time it took for drivers to enter and leave spots [15]. To address this congestion, cities began requiring a minimum amount of off-street parking in their zoning laws [16].

The first major city to require off-street parking was Los Angeles in 1935 [27], and in the following decades, almost all U.S. cities followed. In 1946, 12% of cities zoned for parking, and by 1969, 96% cities with populations over 25,000 followed suit [27]. Authors have asserted that many of these requirements were arbitrary, and if they did rely on empirical data, they prepared for periods of peak rather than typical parking demand [16], resulting in oversupplied parking [16, 28]. Parking-minimum laws for commercial office space, for example, often resulted in roughly 1.5 times as much space to park cars as office space for the employees [16]. In many cities, every 1,000 square feet of restaurant would require 10 parking spaces, meaning that the area of a typical restaurant’s parking lot is often several times larger than that of the restaurant itself [15, 16]. In many locations, this abundant off-street parking is rarely, if ever, fully occupied [16, 28]. Residential parking tends to be oversupplied as well. Grabar describes examples of housing developments where the ratio of required parking spots to apartment units is 3:1 [15]. In some municipalities, the number of required parking spaces exceeds the number of bedrooms. For example, a four-bedroom apartment in Essex, Massachusetts required six parking spots [15], 1.5 per bedroom. Especially in urban areas whose growth has primarily occurred after World War 2 (namely, cities and suburbs throughout the U.S. Sunbelt and suburbs of older, northern U.S. cities), these parking-minimum laws have effectively mandated low building density and car-dependent environments.

## Parking’s Potential Impacts on Health

**Fig. 1:**
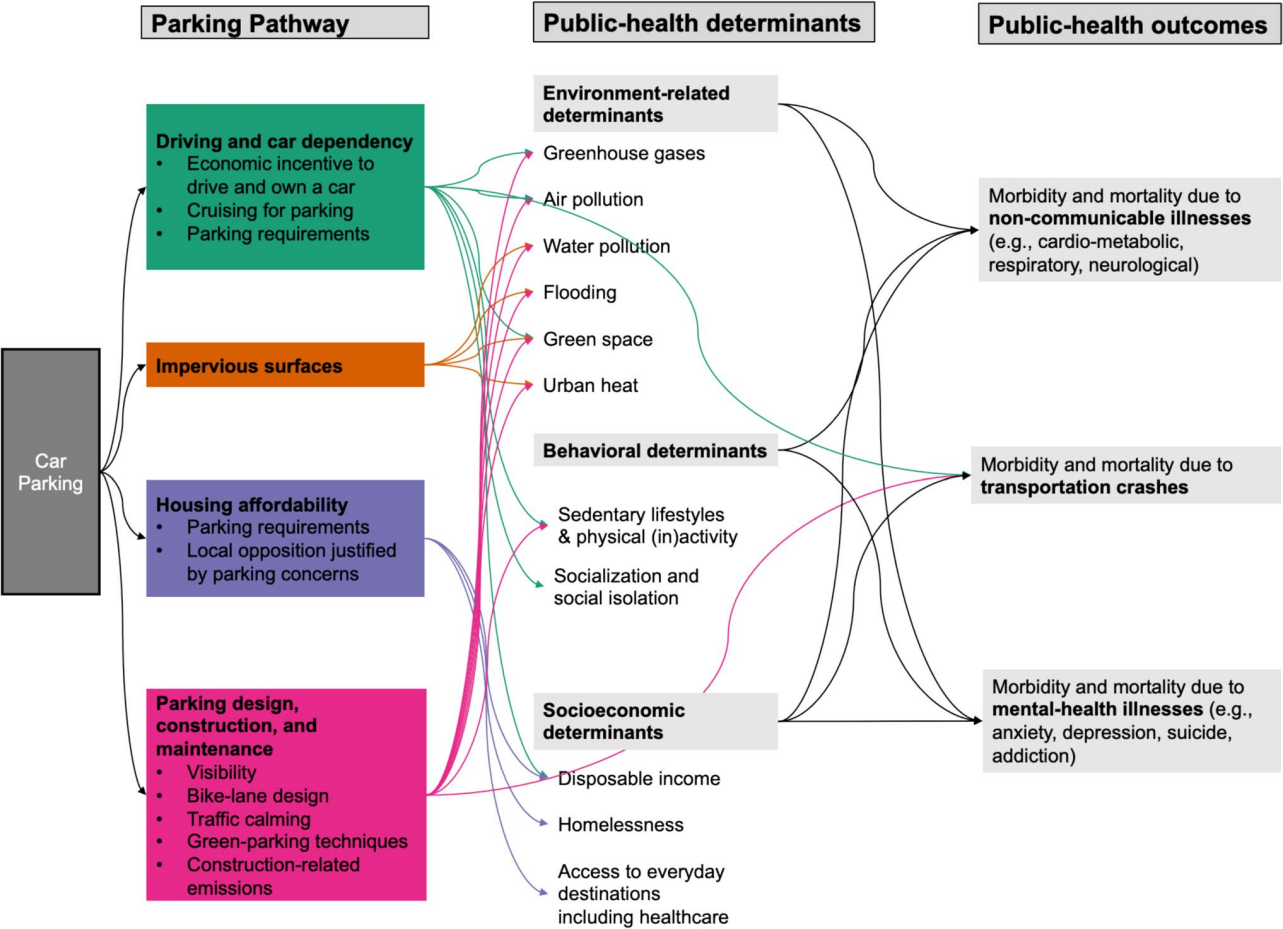
Four pathways through which parking may affect public health

The parking surfaces themselves and the low-density urban form that their abundance has helped to bring about may have several consequences for public health. Informed by Grabar’s chapter [21] and other frameworks relating transportation environments and automobility with health [8, 11], we organize the health impacts of parking as through four principal pathways, illustrated in Fig. 1.

### Promoting Driving and Car Dependency

Perhaps the most important way parking affects public health is by encouraging driving and car-dependent environments. We begin this section by describing the various ways through which parking affects driving and car dependency. We then summarize the health-related consequences thereof.

#### Parking Provision and Driving

Longitudinal evidence suggests the provision of off-street parking encourages more driving. In a study of 9 mid-size cities, each measured at three time points through the 1950s to the early 2000s, authors concluded that an increase in parking provision from 0.1 to 0.5 parking space per person likely caused an increase in automobile mode share of about 30 percentage points [29]. Their longitudinal design, combined with other criteria they considered, led them to conclude that it was more likely that the parking provision caused the driving rather than the converse. The causal relationship between access to parking and more driving may hold regardless of population density. In New York City, the most population-dense U.S. city, authors found that guaranteed access to off-street parking was associated with more driving, even after holding constant population density, access to transit, and socioeconomic measures [30].

#### Underpriced Parking Economically Incentivizes Car Use

One explanation for parking’s apparent positive effect on driving is because it economically incentivizes car use and ownership. Parking’s societal costs tend to exceed its direct costs to motorists both at and away from home [31]. At the non-home destination, parking is free to the driver for an estimated 99% of trips in the U.S [16]. At the home location, parking is often bundled into housing costs [32]. Bundled parking implies that tenants who do not own a car pay the same in rent as those who do park, whether or not they ever use the parking garage for car storage. Of course, this parking is not free to build at either the non-home or home locations [31]. On average, parking costs an estimated $5,000 per vehicle per year, and drivers directly contribute a small fraction of that cost, mostly through home mortgages [31]. The implication is that society at large, including the one third of U.S. individuals for whom driving is not an option (e.g., children, older adults, or those who are blind, disabled, formerly incarcerated, undocumented, or lower income [33]), pay for off-street parking through government spending (i.e., taxes), higher rents, and higher costs for goods and services.

Underpriced parking thus creates a market distortion that incentivizes car ownership and driving [31, 34, 35]. Illustrating the impact of residential parking availability on car ownership and use, a study using a residential lottery showed that random assignment to a home with more parking availability had a positive effect on car ownership and driving frequency [36]. Other studies have similarly found that guaranteed parking at the home is associated with greater vehicle ownership and driving [30, 35, 37].

Underpriced on-street parking in high-demand locations can also cause excess driving, as drivers may “cruise for parking” in search of a spot. Estimates suggest cruising for parking accounts for between 8 and 75% of traffic in high-demand locations [16, 38]. Shoup’s solution for this excess driving is to price the curb parking commensurate with its demand to economically encourage those who must stay in these locations for a longer period of time (such as employees of nearby stores who plan to occupy the space all day) to park in structured parking garages or other off-street spaces to free space for shorter-term visitors who may be willing to pay a higher hourly price [16, 39].

#### Off-street Parking Requirements, Car-dependent Environments, and Driving

Off-street parking requirements are not solely responsible for low-density, car-dependent urban environments in the U.S. Other reasons include subsidized oil, federal highway building, and exclusionary housing zoning. But parking requirements are a major contributor [16, 21]. As described earlier, off-street parking requirements encourage low building density, making it difficult to achieve high-density development with a mix of residential and commercial space. Parking lots often must be larger than the footprint of their corresponding buildings, effectively prohibiting high building density [40]. Whether underground, adjacent to, or a part of buildings, meeting parking requirements adds to construction costs. These costs can be especially high in dense areas with limited space for large parking lots, leading developers to choose instead to build on the outskirts of cities, creating sprawl and low-density communities. As a result, destinations end up being farther apart than they would otherwise be without the oversupplied parking [32].

Low building density, long distances between destinations, and other accompanying attributes of car-dependent environments such as wide roadways and limited pedestrian and bicycling infrastructure, make driving a rational—often the only realistic—choice for those with access to a private car [11]. Using data from the National Household Transportation Survey, a study estimated that moving a household from a suburban to an urban area would reduce its annual car mileage by 18% [41]. Other studies have arrived at similar conclusions: residents of California cities with more compact street networks—the type that rarely coexist with excess off-street parking—drove fewer kilometers per day than those in cities with less dense street networks [42].

#### Health-related Consequences of Driving and Car Dependency

Driving and car-dependent urban planning caused in part by parking and its policy have several negative consequences for public health [8, 9, 11]. First, driving is a major contributor to greenhouse-gas emissions and air pollution [43, 44]. In 2021, transportation accounted for 29% of greenhouse-gas emissions in the U.S., and passenger cars and light-duty trucks contributed (58%) of that 29% [43]. Although the most abundant greenhouse gases (carbon dioxide and methane) do not directly harm human health, their collective accumulation causes climate change and its accompanying health consequences including extreme heat, extreme weather events, climate-sensitive infectious diseases, food insecurity and undernutrition, and population displacement [45]. Air pollution, on the other hand, directly affects human health [46]. Air-pollution emissions from the transportation sector caused an estimated 385,000 deaths globally in 2015 [47]. Second, car crashes are a leading cause of morbidity and mortality, both to car occupants and to other users of the roadway [48–50]. Car-centric environments caused partly by excess parking are more dangerous for people both in and outside of cars [50, 51]. Third, driving is a sedentary behavior, and sedentary behavior is a risk factor for several non-communicable diseases, including diabetes, cancer, and cardiovascular disease [52]. Fourth, abundant parking and its consequences on the urban form make active transportation such as biking and walking unappealing or practically impossible for many trips. Such active transportation could otherwise help individuals meet health-related physical activity guidelines [52, 53].

Fifth and finally, parking and its impact on the built environment can affect accessibility. Accessibility is defined as the ease of reaching destinations [54]. Such destinations include those that are essential for healthcare, employment, or education, along with places for socializing, recreation, and other purposes important for health and wellbeing. For individuals with access to a private car, abundant and free parking can imply accessibility. Some research suggests, for example, that parking fees represent a barrier to health care [55], so free parking may remove this barrier. We caution that this accessibility is limited to those for whom driving is an option, and that this access is unequally distributed in society, as those with more income and wealth are more likely to own a car [56]. The average cost of owning and operating a new car was USD 12,182 in 2022 [57], 16% of median household income [58]. For one fifth of U.S. households, this cost would consume 40% of their income [58]. Less disposable income has health consequences [59, 60]. Beyond the financial burden of car ownership, at the city scale, sprawling development patterns caused partly by parking and parking policy can increase the distance between destinations important for health and wellbeing [11], worsening accessibility, especially for those without access to a car. Parking may confer access, but, as Grabar argues in his conclusion, “it is access of the most superficial sort, one that often papers over deeper inequities” (p. 284 [15]). That is, parking only confers access to some individuals in a world with few alternatives to the car, where destinations are far apart, and where transit is limited or non-existent.

### Creating Impervious Surfaces

Another pathway through which parking, particularly off-street parking, affects health is by occupying expanses of land with pavement or asphalt. This impervious surface has several consequences and opportunity costs for public health.

#### Urban Heat

First is urban heat. At the regional level, cities tend to be hotter than their surrounding rural areas because of their greater land share of heat-absorbing impervious surfaces (e.g., paved parking lots) and less cooling green space [61]. Heat can also vary within urban areas, and areas of the city with more impervious surface tend to be hotter [62]. Urban heat is becoming a more pressing public health concern as the climate warms globally [61], and thus mitigating urban heat is an important public-health priority. Health effects of heat include many outcomes such as heat stroke, heart attack, and acute renal failure [63]. To our knowledge, research has not empirically examined the effects of parking specifically on the heat-health pathway. However, it logically follows that parking infrastructure would contribute to urban heating and thus its health effects, as it occupies a large share of land with impervious surfaces.

#### Flooding

A second health consequence of large areas of impervious surface is their exacerbation of flooding. Flooding can affect human health by displacing residents from homes, causing drowning and injuries, increasing incidence of mental-health concerns such as anxiety and depression, contaminating drinking water supply, and contributing to the spread of communicable diseases [64, 65]. An example of a flooding event exacerbated by impervious surfaces is Hurricane Harvey in Houston, Texas [66, 67]. Between 1992 and 2010, Harris County lost almost 30 percent of its wetlands due to urbanization [68]. This wetland loss was not solely due to parking, but the construction of parking infrastructure was certainly responsible for a considerable share. Multiplying estimates from the Parking Reform Network by 0.75 (as described above) [24], about 20% of the central area of Houston is devoted to parking.

#### Water Pollution

Impervious surfaces also contribute to excess stormwater runoff and water pollution, which can also harm public health [69, 70]. Pavement and asphalt contribute to water pollution in at least two ways. First, the excess runoff they create collects contaminants such as oil, grease, metals, nutrients, bacteria, and sediments and deposits these contaminants in waterways [71]. Second, the materials used for pavement itself are a source of pollution [72].

#### Green Space

Related to the above three topics, an abundance of parking can negatively affect urban green space at multiple scales. Research suggests exposure to nature and green space confers several short- and long-term benefits for human health [73]. It can mitigate the urban heat-island effect, reduce stress, and provide opportunities for physical activity and socializing. Most fundamentally, parking affects green space because a surface parking lot occupies land that was or could be occupied by urban green space. In addition to occupying land that could otherwise be green space where the parking lot itself is constructed, the land-use patterns characterized by abundant parking can cause development to encroach into suburban and peri-urban areas, decreasing green space at the regional scale [74].

Green-parking techniques can mitigate some of these negative health consequences, as we elaborate below.

### Affecting Housing Affordability

#### Parking’s Impact on Housing Costs

Parking may also affect health through its impact on housing affordability and availability. As Shoup [16] and Grabar [15] summarize, parking requirements have made housing more expensive in many areas of the United States and elsewhere. Both statutory parking requirements and local concerns over parking availability by existing residents have limited what types of housing are built, and the housing that is built is more expensive than it would otherwise be were it not for the requirements [32]. For example, after downtown Los Angeles removed minimum parking requirements for new housing, developers included fewer parking spaces than would have been required under the previous law, and, as a result, the total cost of the housing decreased [32]. Gabbe estimates parking comprises 17% of housing costs for renters [40]. As we noted earlier, this cost is commonly bundled into rent [35]. Consequently, carless renters, who, on average, have lower incomes and a higher chance of other structural disadvantages [33], are paying for parking that they may not use. The author estimates that the cumulative “deadweight” cost to these renters is $440 million annually [40].

Parking requirements also affect housing availability and affordability beyond multi-family housing. For example, in 2003, California passed a law allowing accessory dwelling units (ADU, a dwelling on a lot that is not the primary residence). If an ADU was added by converting a garage to housing, however, the law at that time required the parking lost by the garage conversion to be replaced [15]. As a result, few individuals took advantage of this ADU law [15]. Aiming to remove barriers to the housing supply to address California’s housing crisis, in 2019, California removed the requirement that parking lost by a garage-to-housing ADU be replaced [75]. A study on the 2019 revision to the ADU law suggests that it increased the supply and diversity of housing [76].

#### Housing and Health

Higher housing costs attributable in part to parking policy may have several downstream impacts on health. One is homelessness. Homelessness is a major public health issue [77] and is mostly driven by housing affordability [78]. Cities with higher housing costs have more homelessness, even controlling for levels of poverty and other city-level factors commonly thought to be associated with homelessness, such as drug use [79]. Those experiencing homelessness have high rates of chronic mental and physical health conditions, face barriers to accessing medical care, and have higher rates of premature mortality than those with reliable housing [77]. Even if high housing prices do not result in the extreme of homelessness, high housing costs can impact health in many ways [80, 81]. Higher housing prices can cause individuals to compromise on their housing in ways that can impact their health, such as the dwelling’s dimensions, its indoor air quality, and neighborhood characteristics [81]. Furthermore, as housing consumes a larger share of household income [40], less is available for other health-related needs and services. As mentioned, less disposable income tends to lead to worse health [59, 60].

### Parking Design, Construction, and Maintenance

The design, construction, and maintenance of parking can also impact public health both positively and negatively. The design of on-street parking, specifically, may affect visibility and safety for all road users. Meanwhile, green parking techniques present an opportunity for mitigating some health challenges associated with off-street parking. Finally, the construction and maintenance of asphalt can be a non-trivial source of air pollution and greenhouse-gas emissions.

#### On-street Parking and Visibility

One pathway through which the design of on-street parking affects health is its impact on visibility and sight lines. On-street parallel parking near an intersection can obstruct the visibility of drivers, bicyclists, pedestrians, wheelchair users, and other users of the roadway as they navigate the intersection, which could lead to collisions. This visibility obstruction has worsened as vehicles have become larger in the United States [82]. To address this issue, guidance by the National Association of City Transportation Officials (NACTO) recommends removing on-street parking within 20–25 feet of the intersection [83]. This practice, known as “daylighting” [84], increases visibility. A potential negative consequence of removing parking near intersections is that vehicle speeds could rise as drivers may perceive the roadway to be wider (discussed further below) [85]. To improve visibility without encouraging faster speeds, NACTO and others have suggested occupying space that would otherwise be occupied by parking with other features that narrow the roadway, such as bike racks or extra pedestrian space to shorten crossings like curb extensions or bulb-outs [83, 86].

#### On-street Parking and Bike Lanes

The interplay between on-street parking and bike lanes is also important for the safety and comfort of bicyclists. Conventional bike lanes are commonly next to on-street parallel parking. If there is no buffer between the parking lane and the bike lane, the bicyclist riding in the center of that lane will almost certainly be riding in the “door zone”, the area occupied by the opening of a car door, and is at risk of colliding with a car door as it swings open [87]. Dooring accounts for an estimated 12%−27% of urban bicycle-motor-vehicle collisions [87]. One proposed solution to dooring is to add a buffer between the parking lane and the bike lane, moving the center of the bike lane outside of the door zone [87]. Another option is to use angled (diagonal) rather than parallel on-street parking, which would eliminate the risk of dooring bicyclists.

A third solution is to move the bike lane between the on-street parking and the curb, creating a parking-protected bike lane [88]. This option has the advantage of creating a physical barrier between the motor-vehicle lane and the bike lane, improving safety and reducing stress for bicyclists between intersections [88]. However, if the parking is parallel and there is no buffer between the bike lane and the parking, then it remains possible that a bicyclist could be doored by occupants of the non-driver-side of the vehicle. Importantly, this design effectively eliminates the risk that a doored bicyclist would then be run over by moving vehicles [88].

#### On-street Parking and Traffic Calming

Finally, on-street parking may affect health by calming traffic [85]. Traffic calming refers to physical-design measures intended to reduce vehicle speeds with expected safety benefits for all road users [89]. Speed is a major determinant of the risk and severity of motor-vehicle-related collisions [90], so reducing motor-vehicle speeds has important implications for public health [90]. Generally, drivers slow down when the roadway is in reality (or in their perception) narrower [91, 92], and on-street parking can create this effect [85]. It should be noted that there are many other ways to narrow the roadway beyond on-street parking (e.g., chicanes) [89], while avoiding the excess driving caused by underpriced on-street parking. Cities in Japan, for example, prohibit on-street parking but nevertheless have narrow roadways [93].

#### Green Off-street Parking

Off-street parking can be designed to mitigate some of its negative health and environmental consequences through a set of techniques called green parking. Green-parking techniques include reducing the dimensions of parking lots (e.g., designing for average rather than peak demand); converting impervious surfaces to permeable surfaces such as gravel, cobbles, wood mulch, grass pavers, turf blocks, natural stone, or permeable pavements [94]; adding vegetated buffers; and, as the local climate permits, planting large-canopy trees. These approaches can reduce stormwater discharge and improve water filtration, decreasing accompanying health risks of flooding and water pollution; provide shade and mitigate urban heat [95]; and benefit the broader ecosystem.

#### Emissions from Construction and Maintenance

In addition to causing emissions through its effect on driving, parking infrastructure emits air pollution and greenhouse gases via its construction and maintenance [22]. Including indirect and supply-chain effects, Chester and colleagues estimate a plausible range of the emissions of greenhouse gases, volatile organic compounds, and other air pollutants from the construction and maintenance of parking infrastructure corresponding to various scenarios of the total amount of parking infrastructure in the United States [22]. They estimate that the 105 million spaces at the low end of their range contributes 10 annual teragrams of CO2 equivalent, while the 2 billion spaces at their range’s high end contribute 150 annual teragrams of CO2 equivalent, which corresponds to up to 3% of the total U.S. greenhouse gas emissions in 2020 [96]. The authors also estimated parking infrastructure’s contribution to the total life-cycle emissions of various types of motor vehicles. They estimated, for example, that parking infrastructure contributes 25% of the life-cycle missions of particulate matter (PM_10_) of a pick-up truck per passenger-kilometer traveled [22]. These emissions from the construction and maintenance of parking infrastructure have several harmful short- and long-term effects on human health [45, 46].

## Discussion

We have reviewed four pathways through which parking may impact public health. These pathways include its 1) effects on driving and car dependency; 2) coverage of land with impervious surfaces; 3) impacts on housing affordability; and 4) impacts of its design, construction, and maintenance. Parking is a component of the built environment and transportation infrastructure. As such, it can be considered within previously described frameworks relating transportation systems with health [1, 8, 10]. Our review of parking, specifically, reveals health pathways that are not commonly mentioned in these existing frameworks. For example, we found evidence that parking policies affect the affordability of housing, an important social determinant of health. In addition, while urban heat has been noted as a pathway through which the built environment affects health [8], our review of the extent of parking’s land coverage underscores how much parking specifically may contribute to this heat-health pathway.

### Opportunities for Public Health Research and Practice

Many of these proposed pathways from parking to health involve multiple steps in the causal chain and rely partly on deductive reasoning from one step to another. For example, parking is an impervious surface, and if impervious surfaces contribute to heat, and heat affects health, then parking affects health. As another example, if parking affects driving, and if driving affects health, from individual effects on physical activity to community effects on transportation safety to planetary effects on greenhouse gas emissions, then parking affects individual, community, and planetary health. While evidence supports each step of these multi-step causal chains, we are not aware of research that has empirically assessed all steps in one study from parking to health. More empirical research is needed to assess the various ways parking may affect public health.

Specifically, future research might evaluate aspects of the changing parking policy landscape on public health. A parking-reform movement has emerged in the United States, as exemplified by the founding of the Parking Reform Network in 2019 [97]. A focus of this network is advocacy related to repealing parking-minimum laws, which, as we summarized above, have contributed to excess off-street parking. Other policies include market-based pricing approaches for on-street parking [38]. At this writing, Parking Reform Network has documented over 1,400 cites that have instituted parking reforms [98].A similar resource, called the Parking Reform Atlas, documents examples of parking reform worldwide [99]. Among the highest-profile parking policy changes occurred in California in 2022, when Governor Newsom signed legislation banning California localities from requiring parking spaces for new developments built near transit [100]. Future research could evaluate the health impacts of these and other policies. For example, researchers could evaluate the effect of changes in parking policy on community driving levels, urban heat, the housing sector and thus housing-related health outcomes, and transportation-related morbidity and mortality.

Concurrent with this policy movement, the acute need to move commercial activity outdoors during the COVID-19 pandemic motivated alternative uses of the curb. For example, parklets were created to use on-street parking spaces for dining [15]. These and other reimagined purposes for street space (e.g., tactical urbanism [101]) are part of a broader movement to rethink urban environments, reclaiming space from cars for people [102], with expected benefits for public and planetary health [9].

### Parking has a Space in Public Health

Parking is indeed a determinant of population health because of the many ways in which its abundance and policy likely affect public health [103]. As a major component of the built environment, parking fits naturally within existing public health conceptual models. In Frieden’s health-impact pyramid, parking (or parking-related changes) would fit in the second tier from the bottom: “Changing the Context to Make Individuals’ Default Decisions Healthy” [104]. Factors near the bottom of Frieden’s pyramid are those that cannot easily be modified through individual behavior but can have a major impact on public health. Other examples in this tier include clean water and air, elimination of lead and asbestos exposures, and iodization of salt [104].

In another public health framework, parking would be considered a macrosocial factor [105]. As defined by Keyes and Galea, “Macrosocial factors are factors and systems above the level of organization of individuals that may shape population distributions of health and illness” [106]. Examples include political structures, wealth distribution, cultural norms, and, importantly, for this article, “characteristics of the physical and social environment in which populations are embedded.” [106] Parking is a characteristic of the physical environment in which populations are embedded and is thus a macrosocial factor. Moreover, as described above, parking is ubiquitous. Keyes and Galea argue that small changes in ubiquitous macrosocial factors can have a big impact on the distribution of population health [106]. As a ubiquitous macrosocial factor with many health implications, parking warrants attention by public health research and practice.

## Data Availability

No datasets were generated or analysed during the current study.
